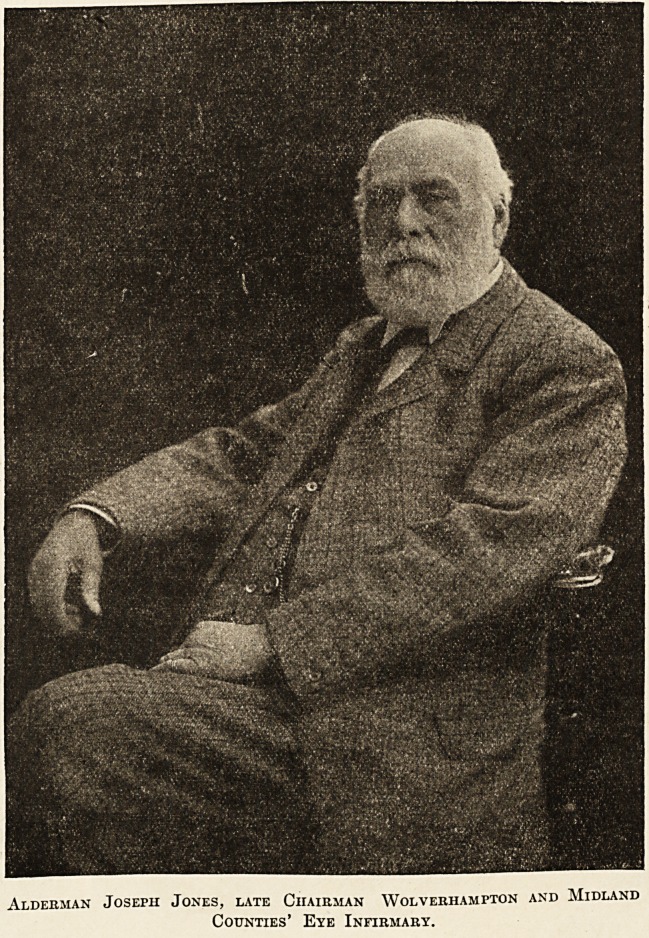# Eminent Chairmen Series
*The previous articles in this series appeared in The Hospital of Oct. 1, Nov. 1, Dec. 10, Jan. 14, Feb. 11, March 11, April 22, May 20, and July 29.


**Published:** 1911-09-09

**Authors:** 


### SPECIAL INSTITUTIONAL ARTICLE.

### EMINENT CHAIRMEN SERIES.*

### X.—MR. ALDERMAN JOSEPH JONES, Late Chairman of the Wolverhampton and Midland Counties' Eye Infirmary.

What follows is written by a late resident who is intimately acquainted with Mr. Jones' work.

The Wolverhampton and Midland Counties' Eye Infirmary was founded in 1881, and has now been established thirty years. For the greater part of that time a member of the Jones family has been on the board of management, and previous to Mr. Joseph Jones' appointment as Chairman in 1897, his brother, Mr. John Jones, held that position. Mr. Jones' success in business is a good example of what might be achieved by any man who possesses perseverance, steadiness of purpose, and a keen practical mind. His great attention to detail and his ability to interest himself in everyone and everything are remarkable.

Alderman Joseph Jones, late Chairman Wolverhampton and Midland Counties' Eye Infirmary.

The Committee took a wise step in 1897 when they appointed him successor to his brother. They realised that his proper appreciation of wealth and his regard for earnest labour would be valuable assets. During the thirteen years that he was Chairman they had every reason to be proud of their selection. He was not merely Chairman of the Board of Management: he was a generous friend to the hospital, and, although he was a member of the Committee of the Wolverhampton and Staffordshire General Infirmary, yet the Eye Infirmary always took priority. His unannounced footsteps were always welcomed, not only by the resident staff, but also by the patients. His generosity was not only exhibited by donations and subscriptions, the total of which would amount to a very large sum, but he would himself bring gifts of eatables and useful articles for the patients and resident staff, while at Christmas and on other festive occasions his thoughtfulness and his regard for detail were nothing short of surprising. He was consulted about everything, and his advice was always definite and practical.

His letter of resignation was received with great regret. He felt that he would not be able to cope conscientiously with the demands on his time and his strength, and that some younger and more energetic member of the Board might take his place. Fortunately for the infirmary he has been persuaded to retain his seat on the Committee. His valuable services and his wide practical experience are thus not lost to the institution.

On the occasion of his retirement in August 1910 the Committee passed the following resolution:

"That the Board of Management of the Wolverhampton and Midland Counties' Eye Infirmary

* The previous articles in this series appeared in The Hospital of Oct. 1, Nov. 1, Dec. 10, Jan. 14, Feb. 11, March 11, April 22, May 20, and July 29.

have received with great regret the letter of resignation of the Chairmanship of the Infirmary from Mr. Alderman Joseph Jones, J.P., and while feeling that they must accept it, they desire to express their appreciation of the services which Mr. Jones has rendered to the infirmary during his thirteen years of office, and of the consistent ability and courtesy with which he has filled the office, and the repeated generosity with which he has treated every need of the institution."

The Committee also decided that the best possible photograph of Mr. Alderman Joseph Jones be obtained and an enlargement of it added to their gallery of photographs of past chairmen.

As noted in the above resolution, Mr. Jones always responded magnanimously to every need of the infirmary, and the same applies to all the other charitable institutions in the town, and Wolverhampton is noted for the large number of the latter. Apart from his office of Justice of the Peace, he is held in great respect by every member of the community. As a large employer of labour in one of the busiest and largest branches of the iron trade in the "Black Country," he has the reputation of kindness, justice, and straightforwardness.

Mr. Jones is a supporter of the ticket system, and during his term of office at the Wolverhampton Eye Infirmary the in-patients increased from 300 in the early years to nearly 700, and the new out-patients from under 4,000 to more than 7,000 in 1910, while at present the yearly attendances border on 18,000.

During this time, too, numerous and extensive improvements have been carried out, in all of which he took the leading part, and gave substantial financial aid. Soon after he took up the reins of office a small addition was made to the building. He has always taken a keen interest in the large and beautiful garden in front of the institution, and from time to time he has given rose trees and numerous plants from his own garden. The out-patient department has practically been rebuilt and brought up to date. Other improvements have also been made in the waiting-hall, the dispensary, the new porter's lodge, etc.

### Personal Interest.

On one occasion when an expensive appliance was needed for the infirmary, and the Committee as a whole could not see their way to sanction it, Mr. Jones, unknown to the Committee, took pains to find out whether the article was really necessary and whether it would increase the efficiency of the institution. When he was satisfied on these points he directed that the house surgeon should obtain the article and send the bill to him. In this way the Eye Infirmary and other charities in the district have benefited on many occasions, and are still benefiting, by his anonymous and unostentatious liberality.

As a Hospital Chairman he may be equalled, but he can hardly be surpassed. Together with his desire for intelligent economy was a keen apprehension of the necessity for keeping appliances up to date so that the efficiency of the institution might be increased, and the working classes should come to look on the infirmary as an immediate resort for first-aid for their eye injuries; all these attributes, and many more, have won for him the reputation of being an ideal Chairman.

## Figures and Tables

**Figure f1:**